# Longitudinal associations of body composition with sleep problems in the first two years after colorectal cancer treatment

**DOI:** 10.1007/s00520-025-10018-6

**Published:** 2025-10-14

**Authors:** Ludovica Margotto, Eline H. van Roekel, Marlou-Floor Kenkhuis, Stephanie O. Breukink, Eric T. P. Keulen, Maryska L. G. Janssen-Heijnen, Ree Meertens, Matty P. Weijenberg, Martijn J. L. Bours

**Affiliations:** 1 Department of Epidemiology, GROW Research Institute for Oncology and Reproduction, Maastricht University, P.O. BOX 616, 6200 MD Maastricht, The Netherlands; 2 Department of Surgery, GROW Research Institute for Oncology and Reproduction, NUTRIM Institute of Nutrition and Translational Research in Metabolism, Maastricht University Medical Centre+, 6229 HX Maastricht, The Netherlands; 3 Department of Internal Medicine and Gastroenterology, Zuyderland Medical Centre Sittard-Geleen, 6162 BG Geleen, The Netherlands; 4 Department of Clinical Epidemiology, VieCuri Medical Center, 5912 BL Venlo, The Netherlands; 5 Department of Health Promotion, Care and Public Health Research Institute (CAPHRI), Institute of Nutrition and Translational Research in Metabolism (NUTRIM), Maastricht University, 6200 MD Maastricht, The Netherlands

**Keywords:** Colorectal cancer survivor, Body composition, Longitudinal, Sleep problems

## Abstract

**Purpose:**

Sleep problems are a frequent concern of colorectal cancer (CRC) survivors. Research on modifiable lifestyle factors that may mitigate sleep problems is sparse. Therefore, we investigated how various body composition parameters are longitudinally associated with sleep problems from 6 weeks up to 24 months post-treatment.

**Methods:**

In a prospective cohort of 396 stage I-III CRC survivors, home-based repeated measurements were conducted at diagnosis and at four post-treatment time points. The insomnia scale of the EORTC QLQ-C30 (range: 0–100) was used to measure sleep problems. Anthropometric measurements of adiposity (BMI, fat percentage, waist-hip ratio) and of muscle mass and muscle function (mid-upper arm muscle circumference, handgrip strength) were employed. Linear mixed models were applied to analyze overall longitudinal associations, and hybrid models were used to disentangle inter- and intra-individual components.

**Results:**

At 6 weeks post-treatment, 47.0% of participants reported sleep problems and symptom severity was at its highest; a decline was observed thereafter. In confounder-adjusted models, no statistically significant overall longitudinal associations of different body composition parameters with sleep problems were found. Intra-individual analyses revealed that increases in BMI were related to less sleep problems over time (*β* per 1 kg/m^2^: -2.8, 95% CI -4.4; -1.2).

**Conclusions:**

BMI increases in the first 24 months post-treatment were associated with decreased sleep problems.

These findings must be interpreted with caution due to the observational design, yet might suggest a potential link between weight regain and sleep problems among CRC survivors recovering from the physical and mental impact of cancer treatment.

**Supplementary Information:**

The online version contains supplementary material available at 10.1007/s00520-025-10018-6.

## Introduction

Globally, colorectal cancer (CRC) represents the third most frequent malignant neoplasm in males and the second in females [1]. In high-income countries, population ageing, socioeconomic development and related lifestyle changes are contributing to a steadily rising incidence [1]. Advances in early detection and treatment are resulting in declining mortality, thus increasing the number of CRC survivors [1–3]. Depending on CRC location, 5-year survival rates for stage I-III range from 53 to 92% [4]. Yet, multiple health-related problems, including sleep problems, and long-term treatment complaints can negatively impact the psychological and physical functioning of CRC survivors [5].

Sleep, regulated in part by interacting homeostatic and circadian mechanisms [6, 7], influences metabolic, endocrine, and immune parameters [8]. Among other controlling factors, sleep architecture coupled with daily energy utilization and storage is controlled by the circadian system [7]. Problems with sleep may encompass a variety of sleep–wake disorders, e.g., sleep-related breathing disorders, such as sleep apneas, or circadian rhythm sleep–wake disorders [9–11]. Yet, the most common sleep problem both in the general population [11] and in cancer populations [12] is insomnia. Sleep problems impair sleep duration and quality, leading to hazardous changes in the glucose-insulin metabolism and substrate oxidation, and in hormone and inflammatory markers concentrations [6–8]. In particular, sleep deprivation reduces glucose tolerance and insulin sensitivity [6–8]. Short sleep also lowers leptin levels and increases ghrelin levels, enhancing appetite and raising the risk of obesity [6–8]. Further, growth hormone secretion decreases, and cortisol levels increase, impairing metabolic homeostasis [6–8]. Finally, sleep problems activate pro-inflammatory pathways, increasing circulating interleukin (IL)-6, tumor necrosis factor (TNF)-α, IL-1β and C-reactive protein, which exacerbate insulin resistance [8].

Specific literature addressing sleep in CRC survivors is sparse. Nonetheless, in two studies of CRC survivors approximately half of the respondents reported symptoms referring to insomnia [13, 14]. Insomnia symptoms include difficulty with sleep initiation or maintenance, frequent awakenings and sleep disturbances, and early-morning awakening with inability to return to sleep, all of which may result from other underlying sleep disorders [11]. Yet, in cancer patients, insomnia may arise from a combination of predisposing, precipitating, and perpetuating factors [12]. For instance, ageing, female sex, and low physical activity predispose for sleep disturbances [12, 15]. Possible precipitants include chemo- and radiotherapy, cancer-related emotional toll and health worry, pain, and treatment and/or surgery functional sequelae (e.g., impaired bowel function and colostomy) [12, 15–17]. Maladaptive sleep practices and attitudes can eventually perpetuate the problem, leading to dysregulated sleep–wake cycles [12]. Thus, CRC survivors can experience sleep problems during and after treatment, hence identifying modifiable lifestyle factors, e.g., body composition, that may contribute to alleviate such difficulties is pivotal.

Increased body fatness, caused by a shift towards high-fat diets and increasingly sedentary lifestyles [18], and a misalignment of feeding rhythms to the sleep–wake cycle [7], has been associated with a greater risk of developing CRC [19]. On a biological level, overweight and obesity are characterized by a low-grade chronic inflammation status, as infiltrating macrophages and adipocytes contribute to raise cytokine concentrations [20]. Further, with ageing, decreased physical activity, and tumor-induced inflammation, muscle mass and muscle quality deplete, resulting in sarcopenia, a disorder which may coexist with obesity (i.e., sarcopenic obesity) [21, 22]. Of CRC patients undergoing treatment, approximately 18 to 61% are viscerally obese, and 16 to 71% are sarcopenic [23], thus excess body weight and muscle wasting are likely to be prevalent conditions in CRC survivors.

The pathophysiological mechanisms linking unhealthy body composition profiles and sleep problems remain unclear, and a vicious cycle has been hypothesized [24]. In the general population, the epidemiological evidence on body composition and sleep suggests a negative association. For instance, cross-sectional associations of short sleep duration (≤ 5 h) and poor sleep quality with higher body mass index (BMI) were demonstrated [25–27]. Likewise, negative cross-sectional associations of sleep duration with waist circumference have been found [28]. Additionally, a meta-analysis of prospective studies in the general population indicated that short sleep duration (≤ 5 or 6 h) was associated with a subsequent development of obesity [29]. However, among cancer survivors, the available research is scarce, limited to cross-sectional study designs, and remains inconsistent. For instance, a study in breast cancer survivors reported that BMI was not cross-sectionally associated with sleep [30]. Similarly, a recent study among CRC survivors reported that sleep duration and sleep quality were not cross-sectionally associated with BMI or waist circumference [31], whereas a cross-sectional study on a mixed cancer types population (of which 20.7% with CRC) concluded that BMIs corresponding to overweight and obesity increased the odds of having poor sleep quality, independently of diet and physical activity [32].

To our knowledge, there are no longitudinal studies evaluating the association between body composition and sleep problems in CRC survivors. Therefore, in the present study we aimed to prospectively investigate how various body composition parameters are associated with sleep problems in CRC survivors from 6 weeks up to 24 months post-treatment. BMI, body fat percentage (estimated using skinfold measurements), and waist-hip ratio (WHR) were used as parameters of adiposity, and mid-upper arm muscle circumference (MUAMC) and handgrip strength were used as parameters of muscle mass and muscle function, respectively. It was hypothesized that increased adiposity and decreased muscle mass and muscle function would be longitudinally related to more sleep problems in the first two years after CRC treatment.

## Methods

### Study design, settings, and participants

The Energy for life after ColoRectal cancer (EnCoRe) study is an ongoing prospective cohort of CRC survivors, initiated in 2012 [33]. Recruitment involves three hospitals in the south-eastern Netherlands, in which patients aged ≥ 18 years old diagnosed with stage I-III CRC are invited to participate [33]. Stage IV CRC patients, patients unable to understand Dutch or not residing in the Netherlands, and patients presenting comorbidities obstructing participation (e.g., Alzheimer’s disease) are excluded [33]. Patients with stage IV CRC were not included in the EnCoRe study, which was set up to investigate the influence of lifestyle on quality of life, because the poor prognosis of patients with metastasized disease, and not lifestyle behaviour, likely determines their quality of life to the largest extent. Upon obtaining informed consent, repeated measurements are performed at diagnosis and at 6 weeks, 6 months, 12 months, and 24 months post-treatment [33]. In the present study, we analyzed available data collected up until July 2018. The EnCoRe study received approval from the Medical Ethical Committee of the University Hospital Maastricht and Maastricht University (Dutch Trial Register number: NL6904; http://www.onderzoekmetmensen.nl/).

### Sleep outcomes

Sleep problems were assessed at all post-treatment time points by using the insomnia scale of the European Organization for the Research and Treatment of Cancer Quality of Life Questionnaire-Core 30 (EORTC QLQ-C30 version 3.0) [32]. The insomnia scale comprises a single item represented by the question “During the past week, have you had trouble sleeping?”, with four response options: “Not at All”, “A Little”, “Quite a Bit”, and “Very Much”. Raw item scores were converted into scale scores (0–100) according to the instructions in the EORTC scoring manual (https://qol.eortc.org/manual/scoring-manual/), with higher scores representing higher levels of sleep problems [32, 34]. Additionally, we identified a “No Sleep problems” category corresponding to the response option “Not at All”, and a “Sleep problems” category corresponding to a merge of the three remaining response options. Clinimetric studies demonstrated that the insomnia scale has a high test–retest reliability (Spearman’s *ρ* = 0.76) [35], can discriminate between patients differing in their clinical status, defined by the Eastern Cooperative Oncology Group performance status scale in terms of self-care, daily activity and physical ability, and is sensitive to change (e.g., scores significantly changed in patients whose clinical status changed) [32].

### Body composition

Body composition was assessed at all time points [33]. During home visits, trained dieticians performed anthropometric measurements according to standard operating procedures [33].

Body weight (kg) was measured using an electronic scale (Seca Ltd, Birmingham, UK type 861), and height (cm) was measured in duplicate (only at diagnosis) using a portable stadiometer (Instrument Development & Engineering, Maastricht University) [36]. BMI (body weight/mean height^2^) was used as a continuous as well as a categorical variable. The World Health Organization (WHO) categorization was applied to classify participants as normal weight (18.5 ≤ BMI < 25 kg/m^2^), overweight (25 ≤ BMI < 30.0 kg/m^2^), and obese (BMI ≥ 30 kg/m^2^) [18]. Skinfold thickness (mm) was measured in triplicate using Holtain skinfold calipers, at the dominant side of the body at four sites (triceps, biceps, subscapular, and suprailiac area) [36]. By applying Durnin-Womersley formulas and Siri’s equation, the sum of the median values of the four skinfold measurements was used to predict body fat percentage [36]. Waist circumference (cm) was measured in duplicate using a circumeter, at the midpoint between the top of the ileac crest and the lower margin of the last palpable rib, and hip circumference (cm) was measured around the widest portion of the buttocks [36, 37]. The WHR (waist circumference/hip circumference) was used as an estimate of fat distribution [37]. A WHR ≥ 0.90 in males and ≥ 0.85 in females were considered as indication of abdominal obesity [37]. The mid-upper arm circumference (MUAC, cm) of the dominant arm was measured in duplicate with a circumeter [36, 38]. The MUAMC (cm), obtained with the formula MUAMC = MUAC − (*π* * triceps skinfold thickness), was used to estimate muscle mass [36, 39]. Maximum handgrip strength (kg) was measured in duplicate with a Jamar dynamometer at the dominant hand, and the highest obtained value considered a proxy of muscle function [36, 40].

### Sociodemographic and clinical characteristics

Data on age (years), sex, and education level (low, middle, high) were self-reported at diagnosis [33]. Current smoking status (yes/no) and having a partner (yes/no) were reported by participants at all time points [33]. Moderate-to-vigorous physical activity (MVPA, hours/week) was determined at all time points with the Short QUestionnaire to ASsess Health-enhancing physical activity (SQUASH) [41]. Participants wore a tri-axial accelerometer for seven consecutive days at all post-treatment time points to determine sedentary time (hours/day) [42]. A seven-day dietary record was collected at all post-treatment time points [33]. Based on the five World Cancer Research Fund/American Institute for Cancer Research (WCRF/AICR) nutritional recommendations regarding fruit, vegetable and whole grain intake, and consumption of ultra-processed foods, red and processed meat, and sugar-sweetened and alcoholic beverages, a continuous dietary quality score (0–5) was calculated [43]. Tumor location (colon/rectum), cancer stage (I, II, or III), stoma (yes/no), chemotherapy (yes/no), and radiotherapy (yes/no) were retrieved from medical records [33]. The Self-Administered Comorbidity Questionnaire was administered at all post-treatment time points to determine the number of comorbidities (0, 1, or ≥ 2) [33, 44]. Fatigue was self-reported at all post-treatment time points with the Checklist Individual Strength (CIS, score range: 20–140; higher score indicates higher level of fatigue) [33, 45]. The EORTC Chemotherapy-Induced Peripheral Neuropathy 20 (CIPN20, score range: 0–100; higher score indicates more symptoms) module was used to assess peripheral neuropathy symptoms at all time points [46].

### Statistical analyses

Descriptive statistics were computed for sociodemographic and clinical characteristics at 6 weeks post-treatment (i.e., the baseline for the longitudinal analyses) [33], and for the outcome at all post-treatment time points, in the total population and in male and female survivors separately.

Linear mixed regression was used for longitudinal analyses [47] from 6 months up to 2 years post-treatment. First, to evaluate whether the development of sleep problems differed between males and females, interaction between sex and time since end of treatment (treated as categorical variable) was explored. Considering the 6 weeks post-treatment time point as reference category, the EORTC QLQ-C30 insomnia scale was regressed on three indicator variables, each representing one post-treatment time point, sex, and their product terms. In separate models, sex-stratified analyses were conducted, and sex differences were tested (male sex as reference category). Next, longitudinal associations between each body composition parameter and the EORTC QLQ-C30 insomnia scale were examined. A first model adjusted for a priori defined confounders including age at enrollment (years), sex, and time since end of treatment (weeks). A second model incorporated additional confounders, identified from the literature and preselected using causal reasoning aided by directed acyclic graphs (DAGs) to identify relevant confounding factors while avoiding overadjustment for mediators or colliders. Variables included current stoma (yes/no), number of comorbidities (0, 1, or ≥ 2), chemotherapy (yes/no), current smoking status (yes/no), MVPA (hours/week), and WCRF/AICR dietary quality score (0–5). Additionally, post-treatment BMI (kg/m^2^) was added to the MUAMC and handgrip strength models only. To explore the potential confounding effect of additional variables, a forward selection approach was used, that is, sedentary time (hours/day), education level (low, middle, high), and having a partner (yes/no) were consecutively added to the models and kept in the models when the change in the regression coefficient of the body composition parameter of interest was > 10% [48]. Ultimately, sedentary time was not included in the handgrip strength model only, education level was excluded from all models, and having a partner was included in all models. Random slopes were tested by means of a likelihood ratio test [47], which indicated that a random slope improved the fit of the handgrip strength model only. Based on regression diagnostics, assumptions were checked and found to not be violated. To disentangle inter- and intra-individual components of the overall longitudinal associations, hybrid models were built [49]. For each subject, the mean value of a body composition parameter over time was used to estimate the inter-individual component, i.e., how differences in each body composition parameter between individuals are associated with sleep problems over time [49]. To the same model, the difference between each subject’s value of a body composition parameter at each post-treatment time point and the subject’s mean value of that body composition parameter over all post-treatment time points (deviation score) was added to estimate the intra-individual component, i.e., how changes in each body composition parameter within individuals are associated with sleep problems over time [49]. Linear-mixed models with standardized exposures [(exposure – exposure mean value)/exposure standard deviation] were fitted to make effect sizes independent of the unit of measurement of each body composition parameter and facilitate comparisons with guidelines for minimal important changes for the EORTC QLQ-C30 insomnia scores [50].

Sleep problems and fatigue are part of a symptom cluster frequently occurring in cancer survivors, and neuropathy and sleep are interrelated [51–53], but the etiology of these complaints [51], and the causal structures in relation to body composition have not been ascertained. Hence, to possibly obtain more insight into these relations, sensitivity analyses were performed by adding fatigue (CIS score, 20–140) to all models, and neuropathy (CIPN20 score, 0–100) to the MUAMC and handgrip strength models. Last, to investigate the temporal direction of the associations, time-lag analyses were performed by modelling each body composition parameter at the 6-week, 6-month, and 12-month post-treatment time point with the 6-month, 12-month, and 24-month post-treatment EORTC QLQ-C30 insomnia scale score, respectively [47].

The analyses were performed with Stata 16.0.

## Results

### Response

**Fig. 1:**
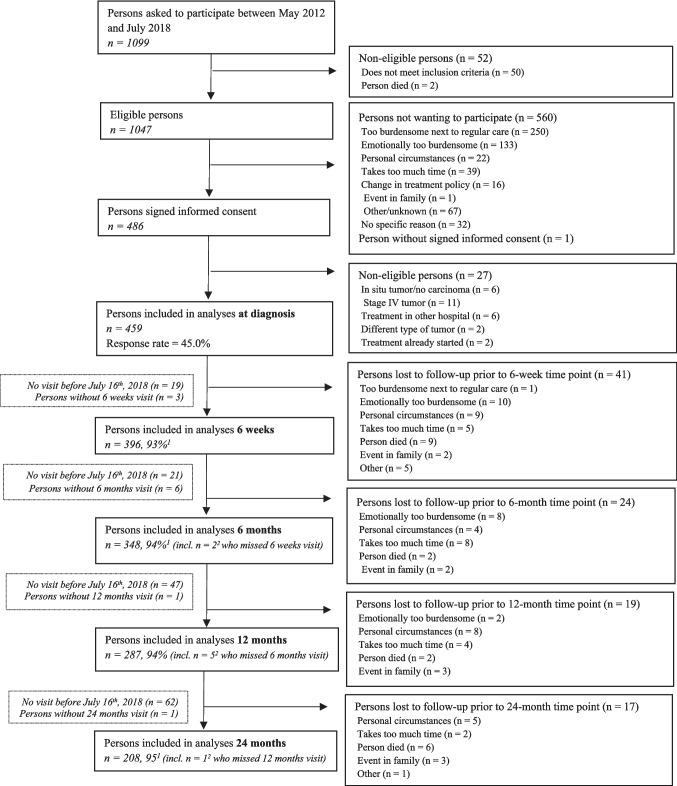
Flow chart of the recruitment and response of participants in the EnCoRe study before July 16th, 2018 [33, 36] ^1^ Response rate post-treatment = (persons included)/(persons included + persons lost to follow-up – persons died) ^2^ One person, out of the three persons without a 6-weeks post-treatment visit, did not have a 6-months post-treatment visit before July 16th, 2018, and one person, out of the six persons without a 6-months post-treatment visit, did not have a 12-months post-treatment visit before July 16^th^, 2018 [36]

In Fig. 1, a flow chart of the recruitment and response of participants up to July 2018 is presented. At diagnosis, the response rate was 45%. Data included 396 CRC survivors who had reached the 6-week post-treatment time point, 348 the 6-month time point, 287 the 12-month time point, and 208 the 24-month time point. The declining number of participants at the consecutive time points is mainly due to the fact that up to July 2018 (i.e., the moment of data freeze) not all participants had reached each time point yet. Response rates were above 90% at all post-treatment time points. Loss to follow-up due to death was below 3% at each follow-up time point.

### Participant characteristics

The characteristics of the total population of CRC survivors, and of male and female survivors separately at 6 weeks post-treatment are presented in Table 1. The mean age was 67 years old (SD 9.1), and 68.2% were males (270/396). The mean WCRF/AICR dietary quality score was 1.9 (SD 0.7), and the median level of MVPA was 7 h/week (IQR 3.5–14.2). Over half of the participants reported ≥ 2 comorbidities (202/395). A total of 250 participants had been diagnosed with colon cancer (63.1%) and the remaining 146 with rectal cancer (36.9%). 31.3% of participants had stage I CRC (124/396), 25.3% stage II CRC (100/396), and 43.4% stage III CRC (172/396). 155 participants had received chemotherapy (39.1%) and 110 had a current stoma (28.3%).

**Table 1:** Sociodemographic, and clinical characteristics at 6 weeks post-treatment of the total population of colorectal cancer survivors and stratified by sex

	Total population (*n* = 396)	Males (*n* = 270)	Females (*n* = 126)
Age (years), *mean* (SD)	67.0 (9.1)	66.7 (8.7)	67.7 (9.8)
Education level (1) ^a^			
Low, *n* (%)	107 (27.1)	58 (21.6)	49 (38.9)
Middle, *n* (%)	149 (37.7)	102 (37.9)	47 (37.3)
High, *n* (%)	139 (35.2)	109 (40.5)	30 (23.8)
Smoking status (yes), *n* (%) (9) ^a^	34 (8.8)	26 (9.9)	8 (6.5)
Having a partner (yes), *n* (%) (8) ^a^	309 (79.6)	226 (85.3)	83 (67.5)
BMI (kg/m^2^), *mean* (SD) (1) ^a^	27.8 (4.6)	27.8 (4.3)	27.8 (5.1)
18.5 ≤ 24.9, *n* (%) ^b^	119 (30.1)	82 (30.4)	37 (29.6)
25.0–29.9, *n* (%)	173 (43.8)	117 (43.3)	56 (44.8)
≥ 30.0, *n* (%)	103 (26.1)	71 (26.3)	32 (25.6)
BMI at diagnosis (kg/m^2^), *mean* (SD) (1) ^a^	28.3 (4.7)	28.3 (4.3)	28.3 (5.5)
Body fat percentage (%), *mean* (SD)	32.9 (6.3)	30.0 (5.0)	38.9 (4.3)
WHR, *mean* (SD) (2) ^a^	0.98 (0.1)	1.01 (0.1)	0.91 (0.1)
< 0.90 ♂ or < 0.85 ♀, *n* (%)	38 (9.6)	13 (4.9)	25 (19.8)
≥ 0.90 ♂ or ≥ 0.85 ♀, *n* (%)	356 (90.4)	255 (95.1)	101 (80.2)
MUAMC (cm), *mean* (SD)	24.7 (2.8)	25.8 (2.5)	22.5 (2.0)
Handgrip strength (kg), *mean* (SD) (2) ^a^	36.3 (11.7)	41.8 (9.2)	24.7 (7.0)
WCRF/AICR dietary quality score (0–5) ^c^, *mean* (SD) (13) ^a^	1.9 (0.7)	1.8 (0.7)	2.1 (0.7)
MVPA (hours/week), *median* (IQR) (6) ^a^	7.0 (3.5–14.2)	9.2 (4.0–16.5)	5.0 (2.0–7.7)
Sedentary time (hours/day), *mean* (SD) (71) ^a^	10.8 (1.8)	11.0 (1.7)	10.5 (1.8)
Number of comorbidities (1) ^a^^,c^, *n* (%)			
None	91 (23.0)	71 (26.3)	20 (16.0)
1	102 (25.8)	78 (28.9)	24 (19.2)
≥ 2	202 (51.1)	121 (44.8)	81 (64.8)
Fatigue (CIS, 20–140), *mean* (SD) (11) ^a^	62.3 (26.2)	61.2 (25.7)	64.5 (27.2)
Neuropathy (CIPN20, 0–100), *median* (IQR) (61) ^a^	3.7 (0.0–13.7)	3.7 (0.0–13.0)	9.2 (1.9–14.8)
Tumor location, *n* (%)			
Colon	250 (63.1)	162 (60.0)	88 (69.8)
Rectum	146 (36.9)	108 (40.0)	38 (30.2)
Cancer stage ^c^, *n* (%)			
I	124 (31.3)	84 (31.1)	40 (31.7)
II	100 (25.3)	66 (24.4)	34 (27.0)
III	172 (43.4)	120 (44.4)	52 (41.3)
Chemotherapy (yes), *n* (%)	155 (39.1)	105 (38.9)	50 (39.7)
Stoma (yes), *n* (%, n) (8)^a^	110 (28.3)	85 (32.1)	25 (20.3)

Abbreviations:* BMI* body mass index, *WHR* waist-hip ratio, *MUAMC* mid upper arm muscle circumference, *WCRF/AICR* World Cancer Research Fund/American Institute for Cancer Research, *MVPA* moderate-to-vigorous physical activity, *CIS* Checklist Individual Strength, CIPN20 EORTC Chemotherapy-Induced Peripheral Neuropathy 20

^a^ Data missing for a few participants (n in brackets)

^b^ There was one person with a BMI below 18.5 kg/m^2^ at 6 weeks post-treatment, having a BMI of 18.2; this person was included in the normal weight category

^c^ The WCRF/AICR dietary quality score includes fruit, vegetable and whole grain intake, ultra-processed foods, red and processed meat, sugar-sweetened and alcoholic beverages [43]

^d^ Because of rounding, percentages do not add up to 100

The mean BMI was 27.8 kg/m^2^ (SD 4.6). According to their BMI, 30.1% of participants had normal weight (119/395), 43.8% overweight (173/395), and 26.1% had obesity (103/395). The mean BMI and distribution across categories were comparable between males and females. The mean body fat percentage was 32.9% (SD 6.3), with males, on average, having a lower body fat percentage than females (30.0%, SD 5.0 versus 38.9%, SD 4.3). 90.4% of participants were classified as having abdominal obesity (356/394); 95.1% of males (255/268) and 80.2% of females (101/126) had a WHR greater than the sex-specific cut-off values. On average, participants had a MUAMC of 24.7 cm (SD 2.8), with males having higher values on average than females (25.8 cm, SD 2.5 versus 22.5 cm, SD 2.0). Similarly, with an overall mean handgrip strength of 36.3 kg (SD 11.7), males showed higher values as compared to females (41.8 kg, SD 9.2 versus 24.7 kg, SD 7.0).

### Sleep problems

Mean scores and frequencies of the EORTC QLQ-C30 insomnia scale at the separate post-treatment time points are presented in Table 2. In the total population, at 6 weeks post-treatment 47.0% of participants reported at least a little sleep problems, with 16.2% reporting quite a bit or very much sleep problems, and the mean insomnia symptom severity score was at its highest (22.5, SD 28.3). Between 6 and 24 months post-treatment, a decrease in mean symptom severity was observed, as well as a decreased frequency of quite a bit or very much self-reported sleep problems (13.1% at 6 months, 13.8% at 12 months, and 12.5% at 24 months). Women were more likely to report quite a bit or very much sleep problems than men (Table 2), and their mean symptom severity showed a different trend over time as compared to their male counterparts, with mean scores increasing until 12 months and decreasing afterwards (Online Resource 1). Treating time as a categorical variable, interaction analysis revealed a statistically significant interaction between time and sex for the EORTC QLQ-C30 insomnia scale between 6 weeks (reference) and 12 months (*p* value 0.013) (Online Resource 2). In sex-stratified analyses, there were statistically significant changes in the EORTC QLQ-C30 insomnia scores in males only (6 weeks vs 6 months *β* −5.4, 95% CI −8.8; −2.1, 6 weeks vs 12 months *β* −6.5, 95% CI −10.1; −2.9). Over time, females scored on average significantly higher than males (*β* 9.7, 95% CI 5.1; 14.4).

**Table 2:** Mean scores and frequencies of the EORTC QLQ-C30 insomnia scale at each post-treatment time point, in the total population of colorectal cancer survivors and stratified by sex

	EORTC QLQ-C30		
	Insomnia scale (0–100)		
	Total *mean* (SD) *response options (*%) ^a^	Males *mean* (SD) *response options (*%) ^a^	Females *mean* (SD) *response options (*%) ^a^
6 weeks (*n* = 389)	22.5 (28.3) 1- 53.0% 2- 30.8% 3- 11.8% 4- 4.4%	20.6 (27.7) 1- 56.8% 2- 29.9% 3- 10.2% 4- 4.1%	26.8 (29.2) 1- 44.7% 2- 35.0% 3- 15.4% 4- 4.9%
6 months (*n* = 343)	19.0 (26.6) 1- 59.5% 2- 27.4% 3- 9.9% 4- 3.2%	15.1 (24.4) 1- 66.7% 2- 23.4% 3- 7.8% 4- 2.2%	26.8 (29.3) 1- 44.5% 2- 35.7% 3- 14.3% 4- 5.4%
12 months (*n* = 283)	19.1 (26.6) 1- 59.4% 2- 26.9% 3- 11.0% 4- 2.8%	13.8 (23.4) 1- 69.4% 2- 21.2% 3- 7.8% 4- 1.6%	30.4 (29.4) 1- 37.8% 2- 38.9% 3- 17.8% 4- 5.6%
24 months (*n* = 199)	19.6 (27.0) 1- 57.8% 2- 29.6% 3- 8.5% 4- 4.0%	16.2 (25.3) 1- 64.7% 2- 25.0% 3- 7.4% 4- 2.9%	27.0 (29.2) 1- 42.9% 2- 39.7% 3- 11.1% 4- 6.4%

Abbreviations:* EORTC* QLQ-C30 European Organization for the Research and Treatment of Cancer Quality of Life Questionnaire

^a^ Percentages of participant responses to the insomnia scale item “Have you had trouble sleeping?”, comprising the response options 1- “Not at all”, 2- “A Little”, 3- “Quite a Bit”, and 4- “Very Much”

### Longitudinal associations of body composition with sleep problems

Age-, sex-, and time-adjusted models yielded statistically non-significant overall associations between each body composition parameter and the EORTC QLQ-C30 insomnia scale, except for WHR (*β* per 0.1 unit: 3.9, 95% CI 1.2; 6.7) (Table 3). In fully adjusted models, we observed a small attenuation of the BMI, MUAMC, and handgrip strength regression coefficients, which remained statistically non-significant; similarly, the WHR estimate was weakened, becoming statistically non-significant. On the contrary, the direction of the association of body fat percentage with sleep problems turned from positive to negative and remained non-significant. In hybrid models, the inter- and intra-individual associations were not significant, except for BMI and MUAMC (Table 3). On average, between 6 weeks and 24 months post-treatment, a 1 kg/m^2^ BMI increase within individuals was significantly associated with a decrease in the EORTC QLQ-C30 insomnia score (intra-individual *β* per 1 kg/m^2^: −2.8, 95% CI −4.4; −1.2). Further, individuals with greater MUAMC values had a higher EORTC QLQ-C30 insomnia score, on average, over time, as compared to individuals with lower MUAMC values (inter-individual *β* per 1 cm: 1.5, 95% CI 0.2; 2.9).

**Table 3:** Longitudinal associations of body mass index, body fat percentage, waist-hip ratio, mid-upper arm muscle circumference, and handgrip strength with the EORTC QLQ-C30 insomnia scale from 6 weeks up to 24 months post-treatment in colorectal cancer survivors

			EORTC QLQ-C30	
			Insomnia scale (0–100)	
			*β*	95% CI
BMI (per 1 kg/m^2^)	Model 1 ^a^	Overall ^l^	0.3	−0.1; 0.8
	Model 2 ^b^	Overall ^l^	0.0	−0.5; 0.6
		Inter-individual ^m^	0.4	−0.2; 0.9
		Intra-individual ^n^	−2.8 ^*^	−4.4; −1.2
	Model 3 ^c^	Overall ^l^	0.0	−0.5; 0.4
	(Sensitivity analysis)	Inter-individual ^m^	0.2	−0.3; 0.7
		Intra-individual ^n^	−2.6 ^*^	−4.2; −1.1
Body fat percentage (per 1%)	Model 1 ^a^	Overall ^e^	0.1	−0.3; 0.5
	Model 2 ^b^	Overall ^l^	−0.2	−0.6; 0.3
		Inter-individual ^m^	0.0	−0.5; 0.5
		Intra-individual ^n^	−0.8	−1.7; 0.2
	Model 3 ^c^	Overall ^l^	−0.2	−0.6; 0.2
	(Sensitivity analysis)	Inter-individual ^m^	−0.1	−0.6; 0.4
		Intra-individual ^n^	−0.5	−1.5; 0.4
WHR (per 0.1 unit)	Model 1 ^a^	Overall ^e^	3.9 ^*^	1.2; 6.7
	Model 2 ^b^	Overall ^l^	1.6	−1.5; 4.7
		Inter-individual ^m^	1.8	−1.8; 5.4
		Intra-individual ^n^	1.1	−4.7; 6.9
	Model 3 ^c^	Overall ^l^	0.9	−1.9; 3.9
	(Sensitivity analysis)	Inter-individual ^m^	0.8	−2.6; 4.2
		Intra-individual ^n^	1.5	−4.3; 7.2
MUAMC (per 1 cm)	Model 1 ^a^	Overall ^e^	0.5	−0.2; 1.3
	Model 2 ^d^	Overall ^l^	0.7	−0.3; 1.8
		Inter-individual ^m^	1.5 ^*^	0.2; 2.9
		Intra-individual ^n^	−0.5	−2.1; 1.1
	Model 3 ^e^	Overall ^l^	0.6	−0.4; 1.6
	(Sensitivity analysis)	Inter-individual ^m^	1.2	0.0; 2.5
		Intra-individual ^n^	−0.6	−2.3; 1.0
	Model 4 ^f^	Overall ^l^	0.3	−0.1; 0.1
	(Sensitivity analysis)	Inter-individual ^m^	1.0	−0.4; 2.5
		Intra-individual ^n^	−0.8	−2.6; 1.0
Handgrip strength ^o^ (per 1 kg)	Model 1 ^a^	Overall ^e^	−0.2	−0.5; 0.0
	Model 2 ^g^	Overall ^l^	−0.1	−0.4; 0.2
		Inter-individual ^m^	0.0	−0.3; 0.3
		Intra-individual ^n^	−0.3	−0.7; 0.2
	Model 3^ h^	Overall ^l^	0.0	−0.8; 1.5
	(Sensitivity analysis)	Inter-individual ^m^	0.0	−0.2; 0.3
		Intra-individual ^n^	−0.1	−0.6; 0.3
	Model 4 ^i^	Overall ^l^	0.0	−0.3; 0.3
	(Sensitivity analysis)	Inter-individual ^m^	0.0	−0.3; 0.3
		Intra-individual ^n^	−0.1	−0.6; 0.4

Abbreviations:* EORTC* QLQ-C30 European Organization for the Research and Treatment of Cancer Quality of Life, *β* beta coefficient, *CI* confidence interval; *BMI* body mass index, *WHR* waist-hip ratio, *MUAMC* mid-upper arm muscle circumference

^a^ Model adjusted for age at enrollment (years), sex, time since end of treatment (weeks)

^b^ Model adjusted for age at enrollment (years), sex, time since end of treatment (weeks), current stoma (yes/no), comorbidities (0, 1, ≥ 2), chemotherapy (yes/no), current smoking status (yes/no), MVPA (hours/week), WCRF/AICR dietary quality score (0–5), sedentary time (hours/day), partner (yes/no)

^c^ Model adjusted for age at enrollment (years), sex, time since end of treatment (weeks), current stoma (yes/no), comorbidities (0, 1, ≥ 2), chemotherapy (yes/no), current smoking status (yes/no), MVPA (hours/week), WCRF/AICR dietary quality score (0–5), sedentary time (hours/day), partner (yes/no), fatigue (CIS, 20–140)

^d^ Model adjusted for age at enrollment (years), sex, time since end of treatment (weeks), current stoma (yes/no), comorbidities (0, 1, ≥ 2), chemotherapy (yes/no), current smoking status (yes/no), MVPA (hours/week), WCRF/AICR dietary quality score (0–5), sedentary time (hours/day), partner (yes/no), BMI (kg/m^2^)

^e^ Model adjusted for age at enrollment (years), sex, time since end of treatment (weeks), current stoma (yes/no), comorbidities (0, 1, ≥ 2), chemotherapy (yes/no), current smoking status (yes/no), MVPA (hours/week), WCRF/AICR dietary quality score (0–5), sedentary time (hours/day), partner (yes/no), BMI (kg/m^2^), fatigue (CIS, 20–140)

^f^ Model adjusted for age at enrollment (years), sex, time since end of treatment (weeks), current stoma (yes/no), comorbidities (0, 1, ≥ 2), chemotherapy (yes/no), current smoking status (yes/no), MVPA (hours/week), WCRF/AICR dietary quality score (0–5), sedentary time (hours/day), partner (yes/no), BMI (kg/m^2^), neuropathy (CIPN, 0–100)

^g^ Model adjusted for age at enrollment (years), sex, time since end of treatment (weeks), current stoma (yes/no), comorbidities (0, 1, ≥ 2), chemotherapy (yes/no), current smoking status (yes/no), MVPA (hours/week), WCRF/AICR dietary quality score (0–5), partner (yes/no), BMI (kg/m^2^)

^h^ Model adjusted for age at enrollment (years), sex, time since end of treatment (weeks), current stoma (yes/no), comorbidities (0, 1, ≥ 2), chemotherapy (yes/no), current smoking status (yes/no), MVPA (hours/week), WCRF/AICR dietary quality score (0–5), partner (yes/no), BMI (kg/m^2^), fatigue (CIS, 20–140)

^i^ Model adjusted for age at enrollment (years), sex, time since end of treatment (weeks), current stoma (yes/no), comorbidities (0, 1, ≥ 2), chemotherapy (yes/no), current smoking status (yes/no), MVPA (hours/week), WCRF/AICR dietary quality score (0–5), partner (yes/no), BMI (kg/m^2^), neuropathy (CIPN, 0–100)

^l^ The beta coefficients represent the overall longitudinal difference in the outcome score over time, including inter- and intra-individual components

^m^ The beta coefficients represent the difference in the outcome score over time between individuals differing in a unit of exposure

^n^ The beta coefficients represent the change in the outcome score over time within individuals per 1 unit of exposure

^o^ A random slope was added to the handgrip strength model

^*^ *p* value < 0.05

### Additional analyses

When adding fatigue (CIS score) to the models, and neuropathy (CIPN20 score) to the MUAMC and handgrip strength models, most regression coefficients reflecting the associations between the body composition parameters and the EORTC QLQ-C30 insomnia score slightly attenuated (Table 3). A 1 kg/m^2^ BMI increase within individuals remained significantly associated with a decrease of 2.6 (95% CI −4.2; −1.1) in the EORTC QLQ-C30 insomnia score (Table 3). In time-lag analysis (Online Resource 3), the regression coefficient reflecting the intra-individual BMI association and the inter-individual MUAMC association with the EORTC QLQ-C30 insomnia scale were weaker as compared to the main results and statistically non-significant (*β* −1.5, 95% CI −3.8; 0.7 and *β* 0.9 95% CI −0.5; 2.4, respectively).

## Discussion

In this study, we investigated longitudinal associations between body composition and sleep problems in stage I-III CRC survivors from 6 weeks up until 24 months post-treatment. In the total population, the severity of sleep problems was at its highest at 6 weeks post-treatment, declined thereafter, and yet remained higher than normative data [54]. As compared to male participants, females were more inclined to suffer from sleep problems and experienced on average more severe symptoms at each post-treatment time point. Although no statistically significant overall association of different body composition parameters with sleep problems was observed, a significant intra-individual association was observed for BMI. Individuals whose BMI increased reported less sleep problems over time. Additionally, an inter-individual association was observed for MUAMC, suggesting that survivors with greater MUAMC reported on average more sleep problems over time. These findings were contrary to our prior hypotheses, yet the BMI result is in line with our previous findings regarding longitudinal associations of body composition with quality of life outcomes. We found that several body composition parameters, including BMI, decreased between diagnosis and 6 weeks post-treatment (mean BMI 28.3 kg/m^2^ at diagnosis and 27.8 kg/m^2^ at 6 weeks post-treatment), potentially reflecting the physical impact of CRC treatment [36]. Thereafter, up until 24 months post-treatment, BMI gradually increased and restored to pre-treatment levels (mean BMI 28.3 kg/m^2^ at 24 months post-treatment). This restoration of body composition, including weight regain after the end of CRC treatment, was shown to be associated with better quality of life outcomes [36]. As previously suggested, these trends may be a response to cancer- and treatment-related alterations of body composition rather than a reflection of an uptake of unhealthy lifestyle behaviors, though this requires further investigation [36, 55].

As this is the first study examining longitudinal associations of various body composition parameters with sleep problems in CRC survivors, a direct comparison with previous literature is hindered by differences in research questions (e.g., reference populations, sleep constructs, the direction of associations) and methodologies (e.g., study designs). In the general population, several cross-sectional studies reported associations of short sleep duration and poor sleep quality with higher BMI and greater waist circumference [25–28]. However, this has not been consistently observed in CRC or other cancer survivors. For instance, among CRC survivors no cross-sectional associations of sleep duration and sleep quality with measures of adiposity were observed [31]. Similarly, increased BMI was not cross-sectionally associated with short sleep in breast cancer survivors [30], but it was found to be a significant determinant of poor sleep quality in cross-sectional data from a mixed cancer types population [56]. Altogether, such findings are in contrast with the unexpected direction of the intra-individual longitudinal BMI association with sleep problems that we observed in the present prospective study. Reasons for these inconsistencies are likely due to the abovementioned sources of heterogeneity. Indeed, with regards to the population under study, CRC and CRC treatment impact body composition [57], leading for instance to muscle wasting via inflammation and to weight loss via improper nutrition due to nausea or pain, and they cause psychological distress [21, 22]. Moreover, sleep problems affect sleep duration and quality, and may arise from underlying sleep disorders [11]. Finally, previous studies among cancer survivors were based on cross-sectional data, assessing body composition parameters and sleep over a variable, often long, time frame after cancer diagnosis. The fact that weight regain was found to associate with decreased sleep problems at an intra-individual level may be partly explained by considering that sleep is likely associated with health-related quality of life (HRQoL) [58]. As mentioned, we previously found in CRC survivors that post-treatment increases in adipose tissue and muscle function, potentially indicative of a recovery from the impact of cancer treatment, were longitudinally associated with better HRQoL and lower fatigue levels [36]. Therefore, our current finding may also be a reflection of a post-treatment recovery period, during which restoration of body reserves includes weight regain and concomitant decreases in sleep problems. Yet, because of sample size, we were unable to conduct subgroup analyses based on BMI categories at diagnosis. Also, a consistent pattern across all body composition parameters with sleep problems was not observed. Hence, we encourage future studies to perform such subgroup analyses in order to investigate whether weight at diagnosis modifies associations, and to further investigate whether CRC survivors regain fat or muscle tissue during recovery. Additionally, with regards to clinical relevance, the regression coefficients were small or trivial when compared with minimal important changes for EORTC QLQ-C30 insomnia scores reported in guidelines [50], even after standardization of the exposures (results not shown). Last, attenuated associations in time-lag analysis suggested that higher BMI at previous time points is not associated with less sleep problems at subsequent time points. Hence, there may be bi-directional effects: sleep problems possibly affect BMI in cancer survivors as indicated in previous research [56], although results have not been consistent [30, 31].

Fatigue and neuropathy were not factored in the main models to prevent biases due to the uncertainty regarding their role in the associations under study (e.g., potential confounding versus mediating factor). In sensitivity analyses, the magnitudes of the associations of the body composition parameters with sleep problems were slightly attenuated, suggesting that fatigue and neuropathy influence such associations, though not strongly. Yet, disentangling causal pathways based on our data remains difficult and warrants further investigation.

Strengths of this study include its prospective design with repeated measurements and high response rates at all post-treatment time points. Moreover, body composition was comprehensively assessed by trained dieticians, thus lowering the risk of differential misclassification bias due to possible measurement errors in self-reported data. From a statistical perspective, linear-mixed models allowed to include in the analyses all available data, independently of participants having reached the 24-month post-treatment time point. Last, adjustment for multiple potential confounders was possible. Nevertheless, there are some limitations. First, this study was observational, hence we cannot be sure about the direction of the associations investigated, nor can we establish causality of the associations. Reverse causation could be a possible explanation for the observed association between increased BMI and decreased sleep problems, as improvements in sleep might contribute to weight gain (e.g., through normalized appetite regulation, restored eating behaviours, and improved mood). Also, as response rate at diagnosis was modest, the cohort may have constituted a healthier sample of CRC survivors with regards to body composition and sleep problems. If so, associations may have been attenuated, and the generalizability of the study results may be compromised. Another limitation is that, although a reliable and valid instrument, a single-item self-reported measurement instrument was used to measure sleep, which limits accurate assessment of sleep quality. Additionally, as sleep was a secondary outcome in the EnCoRe study, there is a possibility of residual confounding due to unmeasured variables (e.g., sleep medications, previous sleep history, sleep–wake hygiene practices).

In conclusion, sleep problems are a frequent concern in stage I-III CRC survivors. As within-person increases in BMI were found to longitudinally relate to less sleep problems from 6 weeks up to 24 months post-treatment, body composition recovery after cancer treatment may prove relevant for decreasing sleep problems in the first two years post-treatment. The findings might highlight a potential role of weight regain in alleviating sleep problems, although the small effect sizes suggest that the clinical significance may be limited. Nevertheless, the findings could help inform adaptations of sleep hygiene strategies for this population and may be most effective as part of a comprehensive approach, such as cognitive behavioural therapy for insomnia, which is the first-line treatment for sleep disturbances in cancer survivors [60]. However, further research is needed. Indeed, despite having observed an association of BMI and sleep problems in a multivariable model, we have not accounted for multiple factors that are involved in the BMI and sleep problems changes observed in CRC survivors after the end of treatment. Studies using more thorough investigations of body composition, e.g., CT scans, and biomarkers may be of support in extending and potentially elucidating the biological and circadian pathways linking body composition and sleep, especially in relation to fatigue. Indeed, for instance, BMI may mediate the association between sedentary time and fatigue [59]. Importantly, for such future investigations, better sleep measurements should also be used to comprehensively assess sleep quality and problems, for instance by means of objective sleep assessments through actigraphy or polysomnography, as self-reported measurements alone are not sufficient to fully assess sleep. Moreover, as weight regain within the first two years post-treatment could reflect a healing response to the cancer and its aggressive treatment [55], it may be possible that, after such recovery period, diet and physical activity influencing body composition assume relevance in the association between weight and sleep, and a higher BMI leads to more sleep problems over a longer post-treatment period. Interestingly, our findings also suggest potential sex-specific patterns, with women reporting more sleep problems overall that peak around 12 months post-treatment, whilst sleep problems among men seem to improve more rapidly in the first year after the end of CRC treatment. To this end, future prospective studies should examine the possible role of body composition in associations of lifestyle behaviours with sleep problems, including potential sex differences. Such evidence may be incorporated in lifestyle recommendations for CRC survivors, allowing to embed sleep problems within a holistic conceptual approach of post-treatment patient care that may contribute to alleviate sleep problems.

## Supplementary Information

Below is the link to the electronic supplementary material.

Supplementary file1 (DOCX 125 KB)

Supplementary file2 (DOCX 25 KB)

Supplementary file3 (DOCX 25 KB)

## Data Availability

Data described in the manuscript, code book, and analytic code will be made available upon reasonable request. Requests for data of the EnCoRe study can be sent to Martijn Bours, Department of Epidemiology, GROW Research Institute for Oncology and Reproduction, Maastricht University, the Netherlands (email: m.bours@maastrichtuniversity.nl).
