# Supplementary material for: Longitudinal associations of body composition with sleep problems in the first two years after colorectal cancer treatment
Source: Support Care Cancer. 2025 Oct 14;33(11):946. doi: 10.1007/s00520-025-10018-6 (PMC12521294; doi:10.1007/s00520-025-10018-6)
Supplement: Supplementary file 1 — Supplementary file1 (DOCX 125 KB) [file 520_2025_10018_MOESM1_ESM.docx]

**Supplementary Information**

Longitudinal associations of body composition with sleep problems in the first two years after colorectal cancer treatment

Supportive Care in Cancer

**Authors**

Ludovica Margotto^1^, Eline H. van Roekel^1^ (ORCID 0000-0001-7758-7426), Marlou-Floor Kenkhuis^1^ (ORCID 0000-0002-4199-4326), Stephanie O. Breukink^2^ (ORCID 0000-0002-5445-4011), Eric T. P. Keulen^3^ (ORCID 0000-0001-6666-8773), Maryska L. G. Janssen-Heijnen^1, 4^ (ORCID 0000-0003-3575-6070), Ree Meertens^5^ (ORCID 0000-0001-8424-9142), Matty P. Weijenberg^1^ (ORCID 0000-0003-1695-4768), Martijn J. L. Bours^1^ (ORCID 0000-0002-5558-1258)

**Author affiliations**

^1^ Department of Epidemiology, GROW Research Institute for Oncology and Reproduction, Maastricht University, P.O. BOX 616, 6200 MD Maastricht, The Netherlands

^2^ Department of Surgery, GROW Research Institute for Oncology and Reproduction, NUTRIM Institute of Nutrition and Translational Research in Metabolism, Maastricht University Medical Centre+, 6229 HX Maastricht, The Netherlands

^3^ Department of Internal Medicine and Gastroenterology, Zuyderland Medical Centre Sittard-Geleen, 6162 BG Geleen, The Netherlands

^4^ Department of Clinical Epidemiology, VieCuri Medical Center, 5912 BL Venlo, The Netherlands

^5^ Department of Health Promotion, Care and Public Health Research Institute (CAPHRI), Institute of Nutrition and Translational Research in Metabolism (NUTRIM), Maastricht University, 6200 MD Maastricht, The Netherlands

**Corresponding author**

Martijn J.L. Bours, Mailing Address: Peter Debyeplein 1, 6229HA, Maastricht, The Netherlands

Email: m.bours@maastrichtuniversity.nl; phone number: 003143882903

**
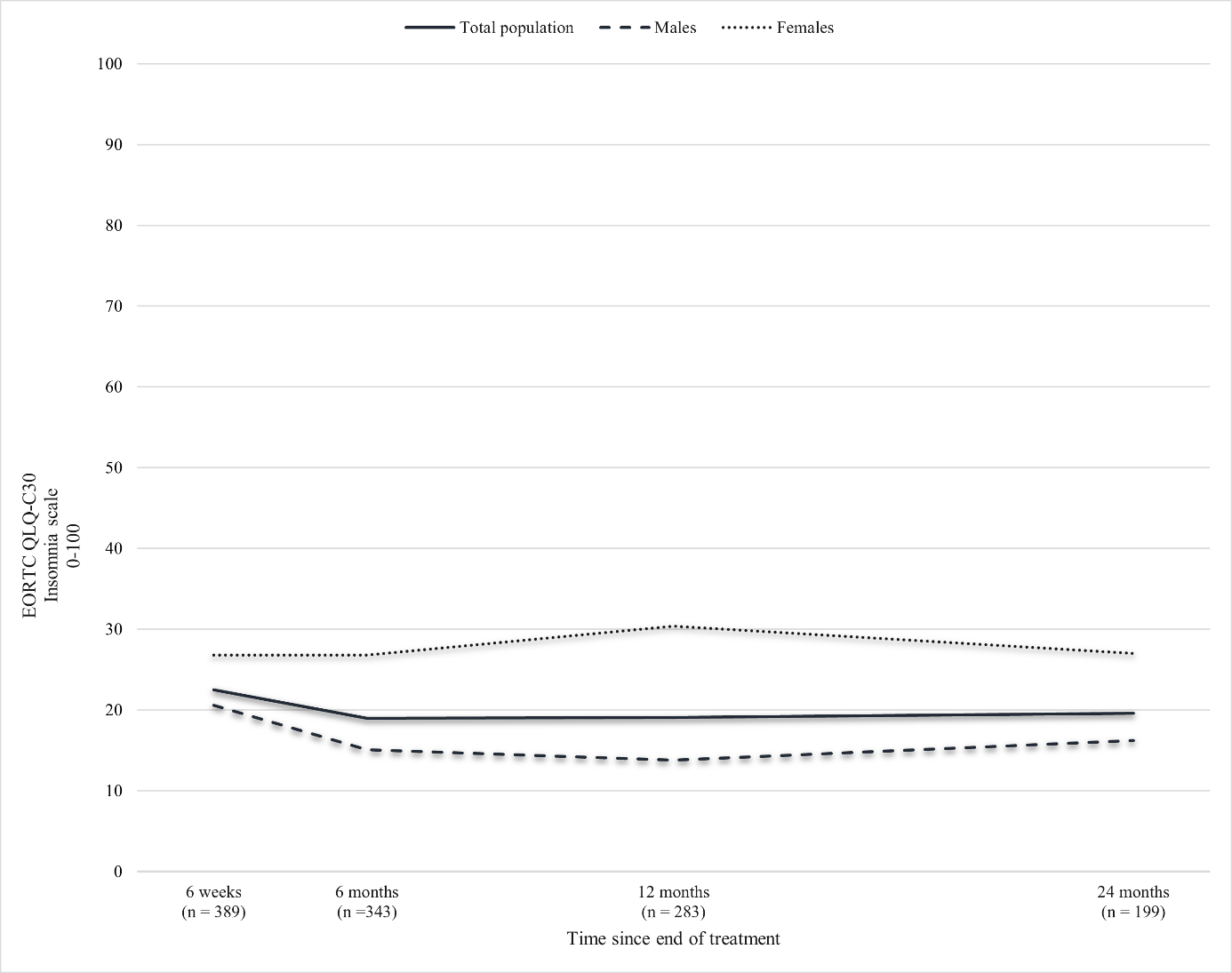
**

**Online Resource 1** Line-graph illustrating mean EORTC QLQ-C30 insomnia scale scores at all post-treatment time points in the total population of colorectal cancer survivors, and by sex

Abbreviations: EORTC QLQ-C30 European Organization for the Research and Treatment of Cancer Quality of Life
