# Supplementary material for: Longitudinal associations of body composition with sleep problems in the first two years after colorectal cancer treatment
Source: Support Care Cancer. 2025 Oct 14;33(11):946. doi: 10.1007/s00520-025-10018-6 (PMC12521294; doi:10.1007/s00520-025-10018-6)
Supplement: Supplementary file 2 — Supplementary file2 (DOCX 25 KB) [file 520_2025_10018_MOESM2_ESM.docx]

**Supplementary Information**

Longitudinal associations of body composition with sleep problems in the first two years after colorectal cancer treatment

Supportive Care in Cancer

**Authors**

Ludovica Margotto^1^, Eline H. van Roekel^1^ (ORCID 0000-0001-7758-7426), Marlou-Floor Kenkhuis^1^ (ORCID 0000-0002-4199-4326), Stephanie O. Breukink^2^ (ORCID 0000-0002-5445-4011), Eric T. P. Keulen^3^ (ORCID 0000-0001-6666-8773), Maryska L. G. Janssen-Heijnen^1, 4^ (ORCID 0000-0003-3575-6070), Ree Meertens^5^ (ORCID 0000-0001-8424-9142), Matty P. Weijenberg^1^ (ORCID 0000-0003-1695-4768), Martijn J. L. Bours^1^ (ORCID 0000-0002-5558-1258)

**Author affiliations**

^1^ Department of Epidemiology, GROW Research Institute for Oncology and Reproduction, Maastricht University, P.O. BOX 616, 6200 MD Maastricht, The Netherlands

^2^ Department of Surgery, GROW Research Institute for Oncology and Reproduction, NUTRIM Institute of Nutrition and Translational Research in Metabolism, Maastricht University Medical Centre+, 6229 HX Maastricht, The Netherlands

^3^ Department of Internal Medicine and Gastroenterology, Zuyderland Medical Centre Sittard-Geleen, 6162 BG Geleen, The Netherlands

^4^ Department of Clinical Epidemiology, VieCuri Medical Center, 5912 BL Venlo, The Netherlands

^5^ Department of Health Promotion, Care and Public Health Research Institute (CAPHRI), Institute of Nutrition and Translational Research in Metabolism (NUTRIM), Maastricht University, 6200 MD Maastricht, The Netherlands

**Corresponding author**

Martijn J.L. Bours, Mailing Address: Peter Debyeplein 1, 6229HA, Maastricht, The Netherlands

Email: m.bours@maastrichtuniversity.nl; phone number: 003143882903

**Online Resource 2** Longitudinal associations of the EORTC QLQ-C30 insomnia scale with time (treated as a categorical variable) from 6 weeks up to 24 months post-treatment and sex in colorectal cancer survivors

|  |  |  | EORTC QLQ-C30 | | | |  |
| --- | --- | --- | --- | --- | --- | --- | --- |
|  |  |  | Insomnia scale (0-100) | | | |  |
|  |  |  | Total population (n = 396) | | | |  |
|  |  |  | Males (n = 270) | | Females (n = 126) | | Interaction ^b^ |
|  |  |  | *β* | 95% CI | *β* | 95% CI | p value |
| Time ^a^ | Model 1 ^c, d^ | 6 months | -5.4 ^*^ | -8.8; -2.1 | 1.0 | -6.2; 4.2 | 0.147 |
|  |  | 12 months | -6.5 ^*^ | -10.1; -2.9 | 1.6 | -4.0; 7.3 | 0.013 ^*^ |
|  |  | 24 months | -3.4 | -7.5; 0.6 | -2.6 | -9.0; 3.8 | 0.803 |
|  |  |  | *β* | 95% CI |  |  |  |
|  | Model 2 ^e, f, g^ |  | 9.7 | 5.1; 14.4 |  |  |  |

Abbreviations: EORTC QLQ-C30 European Organization for the Research and Treatment of Cancer Quality of Life Questionnaire; *β* beta coefficient; CI confidence intervals

^a^ Time refers to time since end of treatment

^b^ p value of interaction term between time (i.e., three indicator variables) and sex, obtained in model with time (i.e., three indicator variables), sex, interaction term

^c^ Model with time (i.e., three indicator variables), stratified by sex

^d^ The beta coefficients represent the mean change in the EORTC QLQ-C30 insomnia scale score over time between each post-treatment time point and the reference in males and females separately

^e^ Model with time (i.e., three indicator variables), sex

^f^ The beta coefficient represents the mean difference in the EORTC QLQ-C30 insomnia scale score over time between males and females

^g^ Male sex is the reference category

^*^ p value < 0.05
