# Supplementary material for: Longitudinal associations of body composition with sleep problems in the first two years after colorectal cancer treatment
Source: Support Care Cancer. 2025 Oct 14;33(11):946. doi: 10.1007/s00520-025-10018-6 (PMC12521294; doi:10.1007/s00520-025-10018-6)
Supplement: Supplementary file 3 — Supplementary file3 (DOCX 25 KB) [file 520_2025_10018_MOESM3_ESM.docx]

**Supplementary Information**

Longitudinal associations of body composition with sleep problems in the first two years after colorectal cancer treatment

Supportive Care in Cancer

**Authors**

Ludovica Margotto^1^, Eline H. van Roekel^1^ (ORCID 0000-0001-7758-7426), Marlou-Floor Kenkhuis^1^ (ORCID 0000-0002-4199-4326), Stephanie O. Breukink^2^ (ORCID 0000-0002-5445-4011), Eric T. P. Keulen^3^ (ORCID 0000-0001-6666-8773), Maryska L. G. Janssen-Heijnen^1, 4^ (ORCID 0000-0003-3575-6070), Ree Meertens^5^ (ORCID 0000-0001-8424-9142), Matty P. Weijenberg^1^ (ORCID 0000-0003-1695-4768), Martijn J. L. Bours^1^ (ORCID 0000-0002-5558-1258)

**Author affiliations**

^1^ Department of Epidemiology, GROW Research Institute for Oncology and Reproduction, Maastricht University, P.O. BOX 616, 6200 MD Maastricht, The Netherlands

^2^ Department of Surgery, GROW Research Institute for Oncology and Reproduction, NUTRIM Institute of Nutrition and Translational Research in Metabolism, Maastricht University Medical Centre+, 6229 HX Maastricht, The Netherlands

^3^ Department of Internal Medicine and Gastroenterology, Zuyderland Medical Centre Sittard-Geleen, 6162 BG Geleen, The Netherlands

^4^ Department of Clinical Epidemiology, VieCuri Medical Center, 5912 BL Venlo, The Netherlands

^5^ Department of Health Promotion, Care and Public Health Research Institute (CAPHRI), Institute of Nutrition and Translational Research in Metabolism (NUTRIM), Maastricht University, 6200 MD Maastricht, The Netherlands

**Corresponding author**

Martijn J.L. Bours, Mailing Address: Peter Debyeplein 1, 6229HA, Maastricht, The Netherlands

Email: m.bours@maastrichtuniversity.nl; phone number: 003143882903

**Online Resource 3** Time-lag analyses of longitudinal associations of body mass index and mid-upper arm muscle circumference with the EORTC QLQ-C30 insomnia scale in colorectal cancer survivors

|  |  |  | EORTC QLQ-C30 | |
| --- | --- | --- | --- | --- |
|  |  |  | Insomnia scale (0-100) | |
|  |  |  | *β* | 95% CI |
| BMI  (per 1 kg/m^2^) | Model 1 ^a^ | Overall ^d^ | 0.5 | 0.0; 1.0 |
|  | Model 2 ^b^ | Overall ^d^ | 0.3 | -0.2; 0.9 |
|  |  | Inter-individual ^e^ | 0.5 | -0.1; 1.0 |
|  |  | Intra-individual ^f^ | -1.5 | -3.8; 0.7 |
| MUAMC  (per 1 cm) | Model 1 ^a^ | Overall ^d^ | 1.0 ^*^ | 0.1; 1.9 |
|  | Model 2 ^c^ | Overall ^d^ | 0.8 | -0.5; 2.0 |
|  |  | Inter-individual ^e^ | 0.9 | -0.5; 2.4 |
|  |  | Intra-individual ^f^ | 0.3 | -1.8; 2.5 |

Abbreviations: EORTC QLQ-C30 European Organization for the Research and Treatment of Cancer Quality of Life Questionnaire; *β* beta coefficient; CI confidence interval; BMI body mass index, MUAMC mid-upper arm muscle circumference

^a^ Model adjusted for age at enrollment (years), sex, time since end of treatment (weeks)

^b^ Model adjusted for age at enrollment (years), sex, time since end of treatment (weeks), current stoma (yes/no), comorbidities (0, 1, ≥2), chemotherapy (yes/no), current smoking status (yes/no), MVPA (hours/week), WCRF/AICR dietary quality score (0-5), sedentary time (hours/day), partner (yes/no)

^c^ Model adjusted for age at enrollment (years), sex, time since end of treatment (weeks), current stoma (yes/no), comorbidities (0, 1, ≥2), chemotherapy (yes/no), current smoking status (yes/no), MVPA (hours/week), WCRF/AICR dietary quality score (0-5), sedentary time (hours/day), partner (yes/no), BMI (kg/m^2^)

^d^ The beta coefficients represent the overall longitudinal difference in the outcome score over time, including inter- and intra-individual components

^e^ The beta coefficients represent the difference in the outcome score over time between individuals differing in a unit of exposure

^f^ The beta coefficients represent the change in the outcome score over time within individuals per unit of exposure.

^*^ p value < 0.05
